# Brain changes in stroke patients during rehabilitation: a longitudinal study

**DOI:** 10.3389/fnins.2025.1636135

**Published:** 2025-07-30

**Authors:** Hongxing Wang, Xuejin Cao, Jia Quan, Hengrui Zhou, Yanli Liu, Wei Wang, Zan Wang, Shenghong Ju, Yuancheng Wang, Yijing Guo

**Affiliations:** ^1^Department of Rehabilitation Medicine, Affiliated Zhongda Hospital of Southeast University, Nanjing, China; ^2^School of Acupuncture-Moxibustion and Tuina, School of Health Preservation and Rehabilitation, Nanjing University of Chinese Medicine, Nanjing, China; ^3^Jiangsu Key Laboratory of Molecular and Functional Imaging, Department of Radiology, Zhongda Hospital, Medical School of Southeast University, Nanjing, China; ^4^Department of Neurology, Affiliated Zhongda Hospital of Southeast University, Medical School of Southeast University, Nanjing, China

**Keywords:** stroke, hemiplegia, motor recovery, multimodal MRI, longitudinal study

## Abstract

**Background:**

Temporal changes in brain structure and function following rehabilitation, and their relationship with positive recovery in stroke patients experiencing hemiplegia, remain unclear. This study used multimodal magnetic resonance imaging (MRI) to investigate the longitudinal changes in the brains of stroke patients with good outcomes after motor rehabilitation.

**Methods:**

Eight subcortical ischemic stroke patients with hemiplegia were enrolled. Multimodal MRI data and clinical assessments were collected in the stable post-acute period and at a 3-month follow-up. Functional connectivity (FC) was calculated for motor-related regions of interest (ROIs) based on functional MRI data. Gray matter volumes (GMVs) and diffusion tensor imaging (DTI) parameters were analyzed to evaluate the temporal changes during recovery.

**Results:**

Compared with initial scans, follow-up scans revealed FC changes between several brain regions, e.g., FC increased between the ipsilesional thalamus and the contralesional middle temporal gyrus (MTG). Increased GMV was observed in the contralesional MTG, while GMV decreased in the contralesional cerebellum, correlating with Action Research Arm Test (ARAT) scores at follow-up.

**Conclusion:**

The findings suggest that MTG is a key area for neuronal activation and functional reconstruction in stroke patients during motor recovery. These results deepen our understanding of the imaging manifestations of structural and functional neural remodeling during rehabilitation.

## 1 Introduction

Ischemic stroke often destroys neurological tissues in the infarcted area, which can result in severe disability. Patients’ long-term outcomes depend on the underlying neural mechanisms that promote post-stroke recovery. Previous studies have found altered functional connectivity (FC) and brain structure during stroke rehabilitation. Resting-state functional magnetic resonance imaging (rs-fMRI) studies typically define the primary motor cortex (PMC) as the region of interest (ROI) for analyzing dynamic FC changes. In one study, the FC between the affected PMC and the contralesional thalamus, supplementary motor area (SMA), and middle frontal gyrus (MFG) was positively correlated with motor recovery 6 months post-stroke (Park et al., 2011). In stroke patients, the FC between the affected PMC and the contralesional sensorimotor cortex, bilateral SMA, and inferior parietal lobe has been observed to transition from lower compared to controls to progressively stronger after 1 month of treatment with multiple rehabilitation interventions, with the potential for normalization of the connectivity level in patients exhibiting good recovery (Golestani et al., 2013). Brain structural imaging studies have found reduced gray matter and ipsilesional striatal and thalamic volumes around lesions in patients with cerebral infarcts in the middle cerebral artery area (Kraemer et al., 2004). White matter fiber degeneration may occur distal to the injury (Yu et al., 2009), and subcortical infarcted areas may induce focal thinning of the ipsilesional frontal cortex through connectivity fibers (Duering et al., 2015; Werden et al., 2017). Secondary damage to motor pathways may be responsible for atrophy of distal gray matter structures.

Several studies have investigated neuroplastic changes in gray matter structures after stroke. Some indicate no change nor a decrease in gray matter (Diao et al., 2017; Fan et al., 2013; Schaechter et al., 2006; Werden et al., 2017), while others demonstrate both decreases and increases (Cai et al., 2016; Cheng et al., 2015; Duering et al., 2015; Jones et al., 2016; Zhang et al., 2014). Some studies have observed an increase in cortical thickness without accompanying atrophy (Liu et al., 2020). In chronic stroke patients who experience good recovery of motor function, evidence has been found of both structural damage and functional reorganization in the affected PMC area (Cheng et al., 2015). Research in stroke patients has also discovered increased gray matter volume (GMV) in the contralesional SMA and increased cerebral blood flow in the contralesional superior frontal gyrus (SFG) and supramarginal gyrus (Miao et al., 2018). These studies suggest that the clinical recovery of patients with hemiplegia is associated with compensation and the remodeling of brain structure and function, with the contralesional cortex playing an important role in the rehabilitation process (Cai et al., 2016).

Due to the long follow-up time and relatively high attrition rate, longitudinal studies of motor recovery in stroke typically analyze individual modalities. Additionally, variations in patient selection criteria and the diverse methods used lead to differences in results across studies. In the present study, we employed multi-model magnetic resonance imaging (MRI) to investigate rs-FC, gray matter structures, and white matter fiber changes in stroke patients with good motor recovery, to identify the neural features that correspond to functional improvement in patients during rehabilitation.

## 2 Materials and methods

### 2.1 Participants

We enrolled eight stroke patients with hemiplegia who had good recovery, each of whom underwent MRI scanning at 1–4 weeks after onset, and 3 months later. The inclusion criteria were as follows: (1) first-onset stroke patients with hemiplegia; (2) no history of neurological or psychiatric disorders; (3) age ≥18 years. All the affected extremities were evaluated for motor function. We used the Fugl–Meyer assessment (FMA) and the Action Research Arm Test (ARAT) to evaluate the motor outcomes of the upper and lower extremities on the affected side.

All eight patients were hospitalized during the acute stage. After completing one course of rehabilitation (approximately 3 weeks), they were discharged and continued limb function exercises on their own. They were followed up and examined 3 months after the initial examination. The rehabilitation training content was aligned with regular hospital procedures, including physical factor therapy (such as medium- and low-frequency electrical stimulation), exercise therapy (joint movement, stretching, neurophysiological therapy), and occupational therapy (primarily training the upper limbs by engaging in daily activities), among others. All rehabilitation treatments were routine and standardized procedures, and no new rehabilitation training (such as transcranial magnetic stimulation, transcranial direct current stimulation, and other brain stimulation) was provided. During the 3 weeks patients spent in the hospital, they received rehabilitation treatment 6 days a week. Each activity was carried out at the same time every day and included 20 min of medium- and low-frequency electrical stimulation, 20 min of upper and lower limb power training, 40 min of one-on-one physical therapy training with the therapist, and 40 min of occupational therapy. Additionally, patients were treated with medication. After discharge, the self-training consisted of items that each patient could complete independently, such as active limb movements, active auxiliary movements of the affected limb, and self-stretching, etc.

At the follow-up stage, the patients demonstrated a significant improvement in motor function, with an increase of more than 10 points in their FMA and ARAT scores (Table 1). All patients provided written informed consent before the examination. The study protocol was approved by the Ethics Committee of Southeast University-affiliated Zhongda Hospital (reference number: ZDSYLL159-P01).

**TABLE 1:** Patient characteristics.

ID	1	2	3	4	5	6	7	8	Mean
Gender	F	F	M	M	M	M	M	M	
Age (years)	48	53	58	34	49	77	56	53	53.5
Lesion side	L	L	L	R	R	R	R	R	
Lesion region	BG	BG	BG	BG	Pons	Pons	BG	Pons	
FMA1	40	28	55	25	51	62	38	25	40.5
FMA2	58	48	73	61	78	96	89	93	74.5
ΔFMA	18	20	18	36	27	34	51	68	34
ARAT1	0	0	29	0	20	40	0	0	11.1
ARAT2	25	19	55	11	54	57	57	57	41.9
ΔARAT	25	19	26	11	34	17	57	57	30.8
1st scan since stroke (weeks)	1	4	2	3	4	1	3	1	2.4
2nd scan since stroke (months)	3.2	4	3.5	3.7	4	3.2	3.7	3.2	3.6

ID, identity document of the 8 enrolled patients; F, female; M, male; R, right; L, left; BG, basal ganglia; FMA, Fugl-Meyer assessment (full score = 100); FMA1 and ARAT1 were assessed at 1st scan (initial); FMA2 and ARAT2 were assessed at 2nd scan (follow-up); Δ was the difference between the second minus the first.

### 2.2 MRI data acquisition

All patients were scanned twice using a 3.0 Tesla Philips (Ingenia) Medical System equipped with a Synergy-L Sensitivity Encoding (SENSE) head coil. Detailed image acquisition procedures are described in the Supplementary material. The first scan was performed at least 1 week after the onset of stabilization. The second scan took place after an interval of 3 months.

### 2.3 Image preprocessing

To pool patients with right- and left-sided lesions together to improve statistical power, the images of patients with right hemisphere lesions were flipped along the midsagittal axis to unify the damaged side. For all patients, we defined the left side as the lesioned hemisphere and the right side as the contralesional hemisphere.

### 2.4 Gray matter volume

The 3D high-resolution T1-weighted images were processed using voxel-based morphometry (VBM)^1^ toolkit SPM8 (Statistical Parametric Mapping, SPM8)^2^. The VBM technique allows for the use of automated post-processed MR images to assess and compare whole-brain GMVs (Ashburner and Friston, 2000). The main preprocessing steps included tissue segmentation, spatial normalization, and modulation to correct for potential volume changes during the spatial normalization step (Good et al., 2001). Then, GMV maps were smoothed using a full width at half maximum (FWHM) kernel of 8 mm and were then used for the subsequent statistical analysis.

### 2.5 Resting-state fMRI analysis

The CONN functional connectivity toolbox (v18.b)^3^ was used to analyze the seed-to-voxel connectivity relationships in the individual patients. Initially, the process involved correcting for slice-timing and bulk-head motion. We used linear regression of the global mean signal, white matter signal, and cerebrospinal fluid signal to remove the effects of nuisance covariates. The images were then normalized to Montreal Neurological Institute (MNI) templates, resampled to 3 mm × 3 mm × 3 mm, and smoothed with a Gaussian kernel (FWHM 8 mm). The temporal signals in the 4-dimensional volume were linearly detrended and band-pass filtered (0.01–0.08 Hz) to remove undesired components.

Subsequently, ROI-wise FC analysis was performed using ROIs identified from the FSL Harvard–Oxford Atlas. Seed ROIs were placed bilaterally in motor system regions in the frontal, parietal, and subcortical areas (including the basal ganglia and thalamus). To compute FC maps corresponding to a selected seed ROI, the regional time course was correlated with all other voxels within the brain. To detect the FC changes in each patient during their rehabilitation, differences between the two scans were analyzed using paired *t*-tests, setting the statistical significance threshold at *p* < 0.001 at the voxel level and *p* < 0.05 at the cluster level (cluster size > 70 voxels), corrected for false discovery rate (FDR).

### 2.6 Tract-based spatial statistics

Diffusion tensor imaging (DTI) analysis was performed using a pipeline toolbox for analyzing brain diffusion images (PANDA)^4^ (Cui et al., 2013). The FMRIB Software Library (Smith et al., 2006) was used to perform tract-based spatial statistics (TBSS). Fractional anisotropy (FA) maps were obtained through preprocessing with an FMRIB Diffusion Toolbox, and the resulting images were included in the TBSS for voxel-wise statistical analysis. After spatial normalization, all FA images were averaged to create a mean FA image, which was then used to generate a skeletonized FA map. Voxel-wise statistical analysis was performed for all voxels with an FA of ≥0.20 to assess differences between the two time points using a paired *t*-test. The Monte Carlo permutation test (5,000 permutations) was used to create the *t*-statistic maps. Statistical images were thresholded at *p* < 0.05, with a family-wise error (FWE) correction for multiple voxel comparisons. The same process was applied to generate statistical maps for mean diffusivity (MD), radial diffusivity (RD), and axial diffusivity (AD).

### 2.7 Statistical analysis

The gray matter images of patients’ acute and chronic phases were statistically analyzed using the SPM-based paired *t*-test to identify brain regions with significant differences (voxel level *p* < 0.001, cluster level > 20 voxels). The results were visualized using xjView software, and the location, size, and coordinates of the brain regions exhibiting differences were recorded. Correlation analysis was performed to investigate the relationship between imaging metrics and behavior, using the Pearson correlation test with a statistical significance threshold set at *p* < 0.05.

## 3 Results

### 3.1 Participants

A total of eight stroke patients were followed up, and brain MRI data and motor function assessments were obtained in both the stabilization phase after acute onset (mean 2.4 ± 1.2 weeks) and in the chronic phase after a 3-month interval with better functional recovery (mean 3.6 ± 0.3 months; Table 1). The injury sites of the eight patients were all in the basal ganglia region or the brainstem (see Supplementary Figure 1).

### 3.2 ROI-wise functional connectivity

**FIGURE 1:**
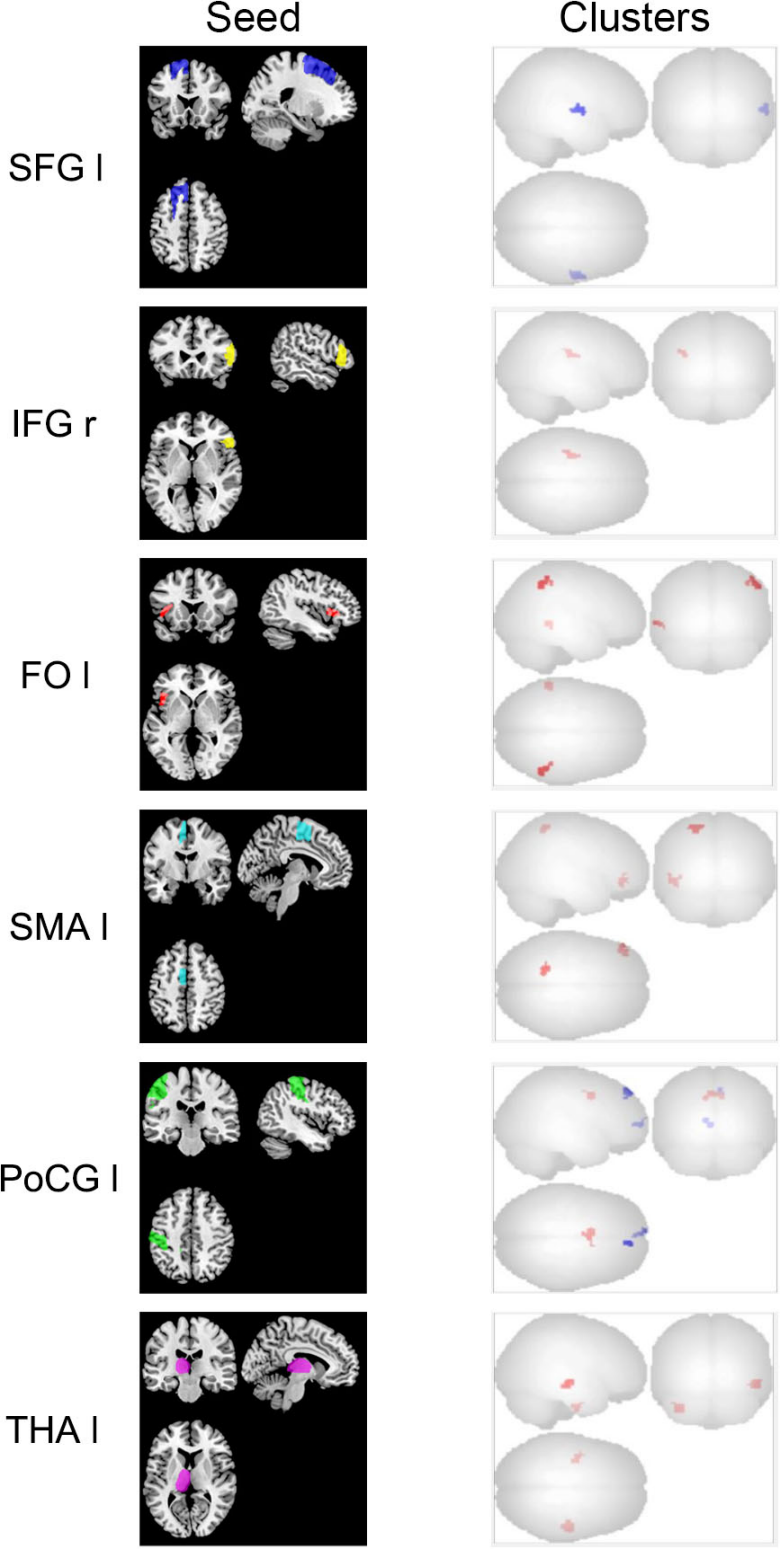
Brain regions with changed rs-FC in stroke patients during the follow-up period. The left column is the seed-ROI diagram. On the right side are the brain clusters with FC changes to each seed-ROI. The clusters are brain areas that have increased (red) or decreased (blue) FC to the seed at the follow-up compared to the initial scan. r, right; l, left; SFG, superior frontal gyrus; IFG, inferior frontal gyrus pars triangularis; FO, frontal operculum cortex; SMA, supplementary motor area; PoCG, postcentral gyrus; THA, thalamus.

The brain regions that exhibited FC changes during the follow-up period included the left superior parietal lobe (SPL), SMA, postcentral gyrus (PoCG), frontal operculum cortex (FO), thalamus, and the right pars triangularis of the inferior frontal gyrus (IFG tri) (Figure 1 and Table 2). Compared with the first examination, the follow-up re-examination of stroke patients showed decreased FC in the left (ipsilesional) SPL and the right (contralesional) superior temporal gyrus (STG). The left SMA demonstrated increased FC to the left frontal pole (FP) and part of the SPL, and the left PoCG showed reduced FC with bilateral FP, increased FC in the anterior cingulate, left paracingulate, and bilateral SMA. The FC increased between the left thalamus and the right middle temporal gyrus (MTG) and the left temporal fusiform gyrus. FC increased between the right pars triangularis of the IFG and the left insular cortex (IC). The left FO showed increased FC to the right angular gyrus (AG), posterior division of the supramarginal gyrus (pSMG), and the left MTG.

**TABLE 2:** Brain regions with changes in FC during follow-up of stroke patients.

Index	Seed	Clusters (MNI coordinate:x,y,z)	Size	*p*-FDR	Color	Areas
1	SFG l	+ 60−12 + 00	149	0.003	Blue	STG r
2	SMA l	−44 + 38−16	123	0.006	Red	FP l
		−20−52 + 60	113	0.006	Red	SPL l
3	PoCG l	−06 + 06 + 42	133	0.005	Red	AC, PaCiG l, SMA
		+ 10 + 50 + 48	85	0.022	Blue	FP r
		−02 + 62 + 02	78	0.022	Blue	FP l
4	THA l	+ 52−18−10	144	0.003	Red	MTG r
		−32−16−42	91	0.017	Red	TFusC l
5	IFG tri r	−32−16 + 24	94	0.024	Red	IC l
6	FO l	+ 54−52 + 50	146	0.001	Red	AG r, pSMG r
		−62−42−04	84	0.014	Red	MTG l

The clusters are brain areas that have increased (red) or decreased (blue) FC to the seed at the follow-up compared to the initial scan. MNI, Montreal Neurological Institute. Voxel size is the number of voxels in the clusters. r, right; l, left. SFG, superior frontal gyrus; STG, superior temporal gyrus; SMA, supplementary motor area; FP, frontal pole; SPL, superior parietal lobe; PoCG, postcentral gyrus; AC, Cingulate Gyrus, anterior division; PaCiG, Paracingulate Gyrus; THA, thalamus; MTG, middle temporal gyrus; TFusC, Temporal Fusiform Cortex; IFG tri, inferior frontal gyrus pars triangularis; IC, insular cortex; FO, frontal operculum cortex; AG, angular gyrus; pSMG, supramarginal gyrus posterior division.

### 3.3 Gray matter volume changes

**FIGURE 2:**
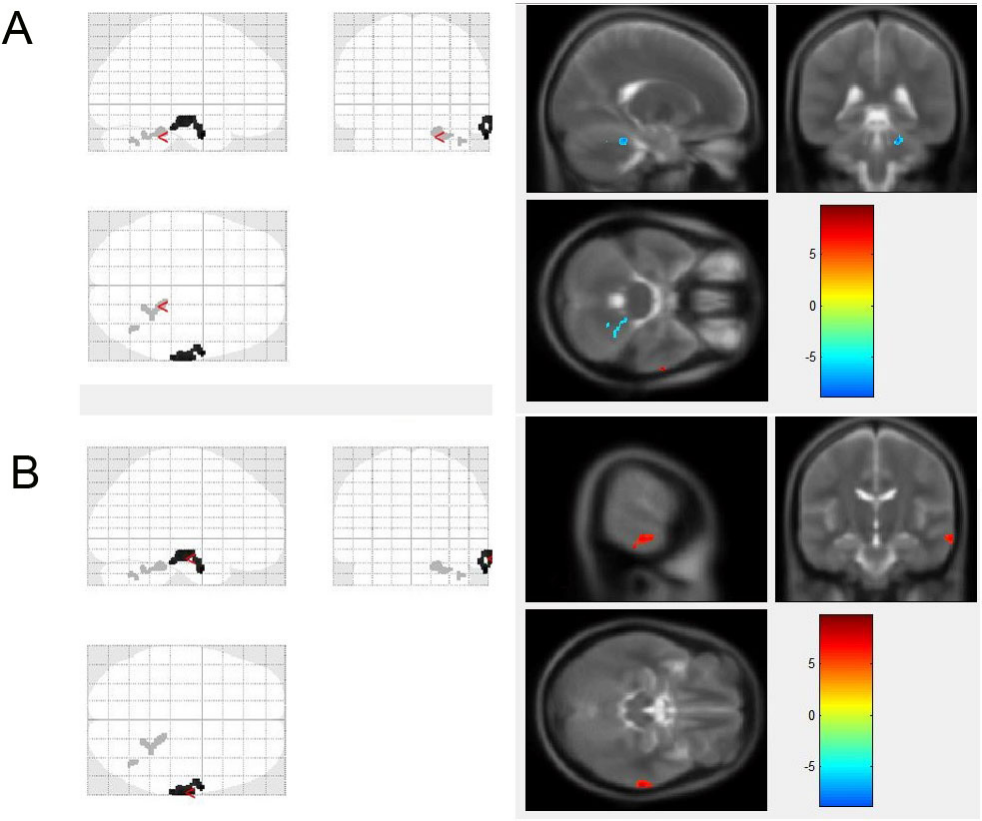
Comparison of GMV in patients after stroke at the initial and follow-up periods. The decreased GMV (blue color) at the follow-up than that at the initial scan of the patients was located in the right cerebellum **(A)**; the elevated GMV (red color) was in the right middle temporal gyrus **(B)**. Color bars are *t*-statistic values from paired *t*-tests.

At the follow-up, compared to the initial scan, stroke patients exhibited elevated GMV in the right MTG, and the reduced mass was located in the right cerebellum (Figure 2 and Table 3). The GMV of the declining cluster was negatively correlated with upper extremity function, as measured by the ARAT score, at the *p* < 0.05 level (*p* = 0.014, *r* = −0.64), and not significantly correlated with the FMA score (*p* = 0.054, *r* = −0.52). The cluster that demonstrated increased GMV did not correlate with motor scores (ARAT, *p* = 0.788, *r* = −0.079; FMA, *p* = 0.578, *r* = −0.162).

**TABLE 3:** Brain regions with GMV changes during the follow-up period in patients.

Index	Change	Peak intensity	Peak MNI coordinate (x,y,z)	Size	Site
1	Decrease	−8.6424	19.5−39−25.5	132	Cerebellum r, anterior lobe
		−5.9516	40.5−63−36	24	Cerebellum r, posterior lobe
2	Increase	9.6255	63−3−31.5	264	MTG r

MTG, middle temporal gyrus; r, right.

### 3.4 White matter alterations

**FIGURE 3:**
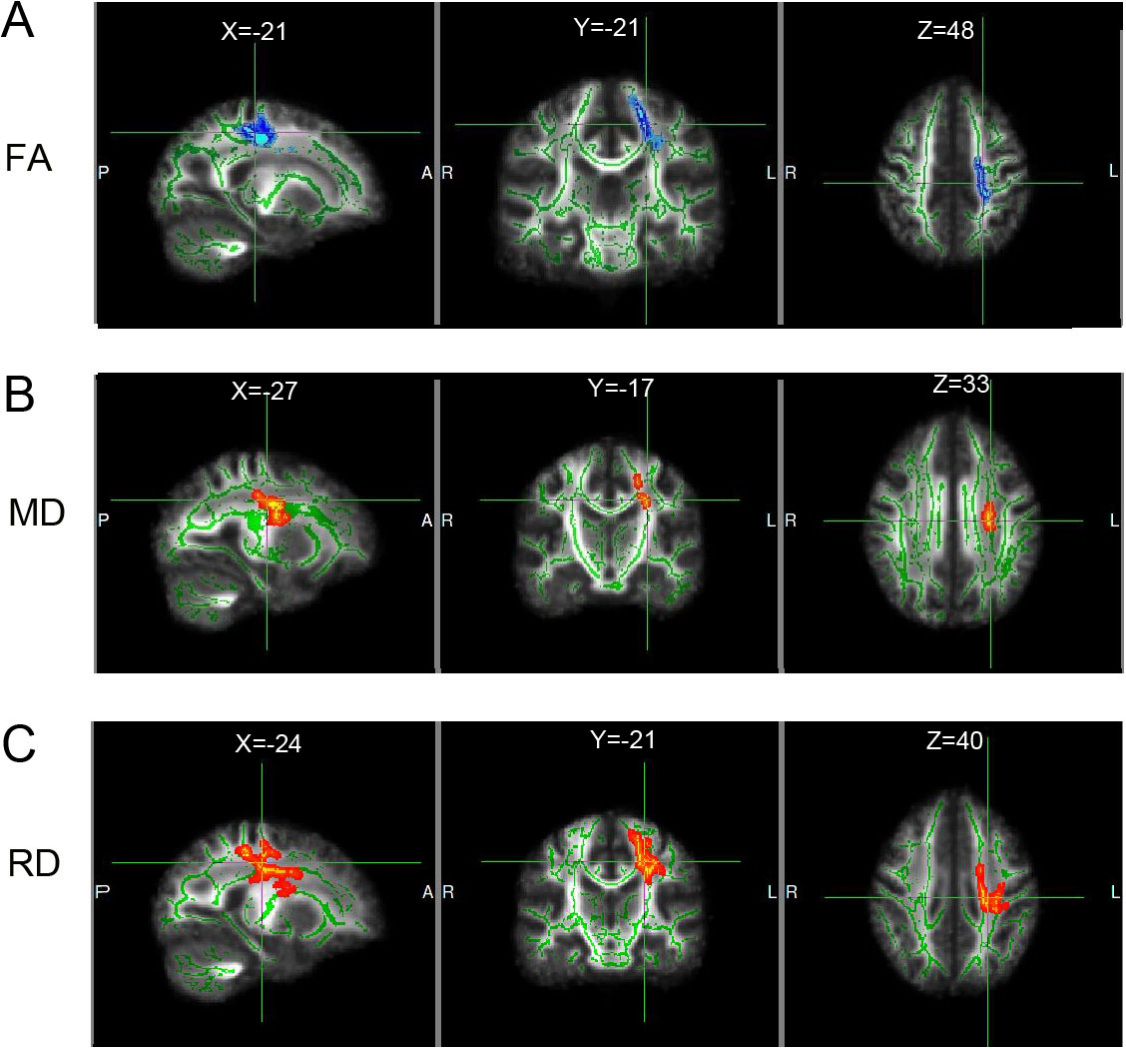
White matter fiber structure that changed during the follow-up period compared with the initial scan. **(A–C)** White matter areas with elevated (red) or decreased (blue) FA, MD, and RD, respectively. Green is the white matter skeleton. Each diffusion tensor index is shown at 1 coordinate point. X, Y, Z are MNI spatial coordinates. L, left, ipsilateral side; R, right, contralateral side.

During the follow-up period, decreased FA was primarily found in the left corticospinal tract (CST) and superior corona radiate (CR) emanating from the left precentral gyrus (PreCG), with a small number of voxels located in the left posterior CR, superior longitudinal fasciculus (SLF), and external capsule, as well as in the left PreCG and paracentral lobule, according to the automated anatomical labeling (AAL) atlas. The sites of increased MD and RD were similar to those of the above fibers (Figure 3). The elevated MD regions were also distributed to the body of the corpus callosum, left PreCG, PoCG, and insula. The location and voxel size for the altered FA, RD, and MD regions are shown in Supplementary Tables 1–3. Mean diffusion tensor values in the clusters did not correlate significantly with motor scores. There were no significant areas of change in AD.

## 4 Discussion

Neuronal plasticity occurs in the early stages post-stroke and can persist for several weeks, involving brain regions distal to the lesion (Cao et al., 2021; Dimyan and Cohen, 2011). Following damage to the cortical motor pathways, neuronal organization of the peripheral brain regions undergoes a gradual process of replacement and remapping of brain tissue, leading to functional reorganization—cortical activation patterns are reestablished, and connections between specific regions are improved during the rehabilitation process (Allegra Mascaro et al., 2019; Feitosa et al., 2023; Volz et al., 2016).

Compared with the first scan, our analysis revealed that the ipsilesional (left) SMA showed elevated FC to the ipsilesional FP and SPL in patients at the follow-up stage. Enhanced connectivity was observed in the left PoCG and bilateral SMA. In our previous research (Cao et al., 2025), we analyzed the overall efficiency of the white matter structure network after stroke and found that it was lower than that of the healthy control group. The local network attribute of the ipsilesional SMA—the node efficiency—was lower than that of healthy individuals. We found that the FA value in this area decreased by TBSS. That is to say, in hemiplegic patients with subcortical cerebral infarction, the ipsilesional SMA brain region is significantly affected by white matter degeneration. In this study, we focused on observing brain changes in patients whose motor function had improved after undergoing rehabilitation exercises, comparing the results before and after rehabilitation. The ipsilesional SMA showed increased FC to the ipsilesional FP and SPL in these patients. We did not compare the patients’ post-recovery MRIs with those of healthy individuals, and thus cannot determine whether this increase in FC exceeds that of those who have not experienced stroke. This issue deserves further in-depth analysis. Regarding the various new rehabilitation techniques that have emerged in recent years, especially non-invasive brain stimulation (NIBS), if they can enhance SMA and FC in the FP and SPL, it is likely to be beneficial for improving motor function.

Functionally, the SMA is involved in the execution of complex and high-accuracy tasks (Azari et al., 1996), whereas the SPL is associated with both the higher-order processing of sensory information and sensorimotor integration, as well as motor learning (Buaron et al., 2020; Leiguarda and Marsden, 2000). These two regions are important as secondary sensorimotor areas, and their enhanced connectivity can positively influence motor recovery when primary motor areas are directly affected by injury. The prefrontal lobe is associated with higher executive functions and decision-related processes. The FP is associated with individual persistence (or degree of adherence) in achieving goals; therefore, effective training can optimize its plasticity, leading to an increase in goal-directed behaviors and the determination to achieve goals (Hosoda et al., 2020). A previous study found increased fALFF in the contralesional parietal lobe, and regional fALFF was correlated with stroke patients’ motor scores compared with controls (Cao et al., 2023). In the present study, we found that the ipsilesional parietal lobe had increased FC with the SMA and FP during the follow-up period in patients with good recovery. It is possible that the link between the ipsilesional parietal lobe, SMA, and FP was enhanced because the patient’s rehabilitation process required the synchronized coordination of motor learning, task execution, and the ability to persist with the training. Persistence in accomplishing goals is particularly important in rehabilitation and often affects the patient’s final outcome, representing a more volitional quality. The reduced FC between the PoCG and the bilateral FP is likely because sensory processing and goal adherence during training are not synchronized—the sensations during training may be unpleasant, while adherence to training requires the patient to ignore the sensations and rely on subjective volition. Furthermore, patients with voluntary motor dysfunction may exhibit corresponding abnormal sensory modulation, as the actions modulate the perception and neural representation of their consequences (Buaron et al., 2020).

The post-stroke relationship between structural and FC is complex [31] (Suarez et al., 2020), with functional brain activity compensating somewhat for structural damage (De Vico Fallani et al., 2017; Liu et al., 2015). For example, in patients with good recovery, a decrease in cortical thickness in the ipsilesional PMC is accompanied by neural activation and higher FC with other brain regions (Zhang et al., 2014). Focal thinning of the ipsilesional frontal cortex can be caused by connective fibers to the infarcted area after subcortical stroke (Duering et al., 2015; Werden et al., 2017). Conversely, an increase in cortical thickness of the contralesional prefrontal lobe, FP, and MTG during recovery occurs within 6 months after stroke (Cai et al., 2016). This increase in cortical thickness is not associated with an improvement in FMA motor scores, however (Fan et al., 2013; Liu et al., 2020). In this study, the elevated GMV in stroke patients at follow-up was also located in the contralesional MTG. The FC between the ipsilesional thalamus and the contralesional MTG increased, so the MTG was presumably a key area of neuronal activation and functional reconstruction in patients during rehabilitation. GMV was reduced in the contralesional cerebellum and correlated with patients’ ARAT scores at follow-up. GMV reduction in the contralesional cerebellum after stroke has also been reported in other research (Jiang et al., 2017; Yu et al., 2017). In a previous study, certain parts of the cerebellum within the SMN displayed reduced FC compared to controls post-stroke, with increased fibers passing through this area (Cao et al., 2023). As rehabilitation progressed, the GMV of the region decreased, possibly as a result of white matter remodeling.

Our TBSS results showed that reduced FA and increased MD and RD were distributed in regions of the CST, superior CR, and SLF during the follow-up stage. These longitudinal white matter alterations were suggestive of direct axonal damage by lesion and glial changes (Boucher et al., 2023; Oey et al., 2019; Umarova et al., 2017). The lack of changes in AD implies a primary involvement of the periaxonal environment rather than the axons, which are involved in structural connectivity changes (Song et al., 2002). Structural changes in distal pathways not directly linked to the lesion represent transneuronal alterations that can only be characterized longitudinally, including neuronal shrinkage, a decrease in dendritic and synaptic numbers, and alterations in axonal myelin and fiber numbers, which correspond to the increased RD (Fornito et al., 2015). One prior study emphasized that not all fibers degenerate and that longitudinal white matter remodeling could be captured in stroke patients suffering neglect, e.g., in the bilateral cingulum (Umarova et al., 2017). Similarly, our previous study reported increased inter-hemispheric connections in stroke patients compared to controls (Cao et al., 2023), suggesting that white matter remodeling could occur during the chronic phase of stroke.

Identifying the phenotype of recovery after stroke and its unique neural basis is the focus of most current functional imaging research. A detailed analysis of changes in connectivity patterns may contribute to a better understanding of brain adaptation following stroke. The occurrence of structural changes throughout the cortex, including frontal and temporal lobe regions, suggests that these sites may be potential targets for neuromodulation such as NIBS techniques (Keser et al., 2023). These regions exhibit significant structural changes during recovery, and modulating them may lead to plastic changes in certain important cortical striatal pathways, thereby favoring motor function recovery. This hypothesis remains to be tested in future clinical trials.

### 4.1 Limitations

This study had multiple limitations, most notably its small sample size and the absence of a control group. However, we dynamically observed changes from the acute to chronic phases, and our statistical methods, including the paired *t*-test (FDR correction), limit many conditions, ensuring the scientific rigor of our findings. In many similar studies, comparisons between stroke and control groups often yield no particular findings regarding individual functional recovery, and their conclusions vary. Due to the influence of various factors, such as the location of the stroke, the type of injury, and the course of the disease, many previous research findings have not been reproducible. For example, the larger a study’s sample size is, the more complex the types of injuries it involves. If the objective of having a small sample size is to minimize sample heterogeneity, expanding the number of patients within a short period of time is challenging. We hope to continue our long-term exploration of topics in this field and conduct future high-quality research to achieve a satisfactory result. Future studies should aim to further explore these findings by expanding the sample size.

## 5 Conclusion

This study used multimodal MRI to explore the temporal changes in brain structure and function in stroke patients during their rehabilitation. At follow-up, patients with good recovery demonstrated FC changes between specific sensorimotor system areas, the coexistence of gray matter atrophy and plasticity, and white matter microstructural changes. Research on the neuroplasticity mechanisms involved in post-stroke recovery has the potential to advance stroke rehabilitation by informing the development of novel treatment strategies.

## Data Availability

The original contributions presented in this study are included in this article/Supplementary material, further inquiries can be directed to the corresponding authors.

## Footnotes

1
http://dbm.neuro.uni-jena.de/vbm8/

2
https://www.fil.ion.ucl.ac.uk/spm/software/

3
https://www.nitrc.org/projects/conn

4
http://www.nitrc.org/projects/panda
