# Supplementary material for: Brain changes in stroke patients during rehabilitation: a longitudinal study
Source: Front Neurosci. 2025 Jul 30;19:1636135. doi: 10.3389/fnins.2025.1636135 (PMC12343724; doi:10.3389/fnins.2025.1636135)
Supplement: Supplementary file 1 [file Data_Sheet_1.docx]

**Supplementary Material**

**Methods**

**Imaging Acquisition**

The subjects were scanned using a 3.0 Tesla Philips (Ingenia) Medical Systems equipped with a Synergy-L Sensitivity Encoding (SENSE) head coil at the ZhongDa Hospital Affiliated to Southeast University. All participants lay supine with the head snugly fixed by a belt and foam pads to minimize head movement. Resting-state functional images, including 240 volumes, were acquired by a echo planar imaging (EPI) sequence: repetition time (TR) = 2000 ms; echo time (TE) = 35 ms; flip angle (FA) = 90°; matrix = 64 × 64; field of view (FOV) = 230 × 230mm2; thickness = 3.6 mm; number of slices = 33.

DTIs were acquired using 33 diffusion-weighted images (b=1000 s/mm2) and a reference T2-weighted image with no diffusion weighting (b=0 s/mm2): voxel size =2×2×2 mm3, gap=0 mm; TE = 107 ms; TR = 5835 ms; FOV = 256 × 256 mm2 ; FA= 90°; matrix = 128 × 128; slices=75.

For the T1-weighted images, parameters included TR = 9.6 ms;TE = 3.7 ms; FA = 9°; acquisition matrix = 256 × 256; thickness = 1.0 mm; number of slices = 140. Additionally, sagittal fluid attenuated inversion recovery (FLAIR) images were obtained with the following parameters: TE = 110 ms, TR = 7000 ms, TI = 2200 ms, flip angle= 90°, matrix size = 480 ×480, thickness = 5 mm, slices= 20.

**Results**

**Participants**

**
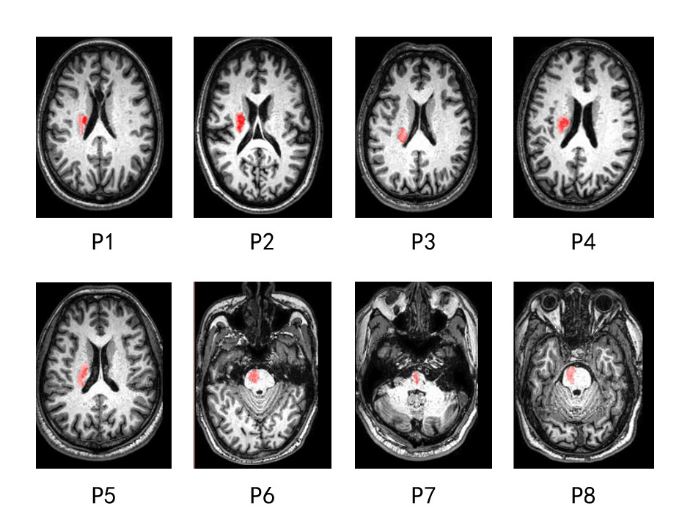
**

**Fig. S1.** The injury sites of the eight patients were all in the basal ganglia region or the brainstem.

**The brain regions with changed diffusion tensor values in the atlas**

Note: voxel size: number of voxels in the cluster. Cluster Locater in PANDA software was used to locate the cluster image according to JHU ICBM-DTI-81White-Matter Labels (White-Matter Tractography Atlas) and automated anatomical labeling (AAL) atlas. White matter atlas (voxel size): the atlas regions this cluster involves and the quantity of voxels in this cluster overlapped with each atlas region. L, left, ipsilateral side; R, right, contralateral side.

**Table S1.** The location of FA decreased regions in the atlas.

White-Matter Tractography Atlas

| Index | voxel size in total | region name | voxel size |
| --- | --- | --- | --- |
| 1 | 499 | Superior.corona.radiata.L | 89 |
|  |  | Posterior.corona.radiata.L | 11 |
| 2 | 291 | Superior.corona.radiata.L | 256 |
|  |  | Posterior.corona.radiata.L | 12 |
|  |  | Superior.longitudinal.fasciculus.L | 4 |
|  |  | External.capsule.L | 2 |

AAL Atlas

| Index | voxel size in total | region name | voxel size |
| --- | --- | --- | --- |
| 1 | 499 | Precental gyrus L | 2 |
|  |  | Paracentral lobule L | 2 |
| 2 | 291 | Not in the atlas |  |

**Table S2.**The location of MD increased regions in the atlas.

White-Matter Tractography Atlas

| Index | voxel size in total | region name | voxel size |
| --- | --- | --- | --- |
| 1 | 283 | Superior.corona.radiata.L | 233 |
|  |  | Posterior.corona.radiata.L | 12 |
|  |  | External.capsule.L | 8 |
|  |  | Superior.longitudinal.fasciculus.L | 4 |
| 2 | 38 | Superior.corona.radiata.L | 23 |

**Table S3.**The location of RD increased regions in the atlas.

White-Matter Tractography Atlas

| Index | voxel size in total | region name | voxel size |
| --- | --- | --- | --- |
| 1 | 1663 | Superior.corona.radiata.L | 706 |
|  |  | Superior.longitudinal.fasciculus.L | 197 |
|  |  | Posterior.corona.radiata.L | 63 |
|  |  | External.capsule.L | 29 |
|  |  | Body.of.corpus.callosum | 15 |

AAL Atlas

| Index | voxel size in total | region name | voxel size |
| --- | --- | --- | --- |
| 1 | 1663 | Precental gyrus L | 21 |
|  |  | Insula L | 5 |
|  |  | Postcentral gyrus L | 2 |
